# SPPS: A Sequence-Based Method for Predicting Probability of Protein-Protein Interaction Partners

**DOI:** 10.1371/journal.pone.0030938

**Published:** 2012-01-26

**Authors:** Xinyi Liu, Bin Liu, Zhimin Huang, Ting Shi, Yingyi Chen, Jian Zhang

**Affiliations:** Department of Pathophysiology, Key Laboratory of Cell Differentiation and Apoptosis of Chinese Ministry of Education, School of Medicine, Shanghai Jiao Tong University, Shanghai, China; Deutsches Krebsforschungszentrum, Germany

## Abstract

**Background:**

The molecular network sustained by different types of interactions among proteins is widely manifested as the fundamental driving force of cellular operations. Many biological functions are determined by the crosstalk between proteins rather than by the characteristics of their individual components. Thus, the searches for protein partners in global networks are imperative when attempting to address the principles of biology.

**Results:**

We have developed a web-based tool “Sequence-based Protein Partners Search” (SPPS) to explore interacting partners of proteins, by searching over a large repertoire of proteins across many species. SPPS provides a database containing more than 60,000 protein sequences with annotations and a protein-partner search engine in two modes (Single Query and Multiple Query). Two interacting proteins of human FBXO6 protein have been found using the service in the study. In addition, users can refine potential protein partner hits by using annotations and possible interactive network in the SPPS web server.

**Conclusions:**

SPPS provides a new type of tool to facilitate the identification of direct or indirect protein partners which may guide scientists on the investigation of new signaling pathways. The SPPS server is available to the public at http://mdl.shsmu.edu.cn/SPPS/.

## Introduction

The molecular network maintained by different types of protein interactions is widely manifested as the fundamental driving force of cellular operations [1]. Crosstalk between proteins instead of individual components leads to many biological functions [2]. Therefore, different means of discovering protein partners in the global network have been considerably valued since these are required to address the imperative principles of biological systems [3]. However, the general methodology for searching protein interaction partners in the genomes, such as large-scale yeast two-hybrid approaches or coimmunoprecipitation methods [4], is time-consuming and expensive, especially in the high-throughput mode. Therefore, a universal computational tool, which can provide an expeditious way for the recognition of potential protein interacting partners *in silico*, is favourable to enhance the efficiency on the investigations of new signaling pathways.

Computational methods for protein-protein interaction (PPI) prediction are based on protein sequence, structural and genomic features that related to interactions and functional relationships [5]–[7]. Such methods include phylogenetic profile [8]–[10], phylogenetic tree [11], gene neighbor and gene cluster methods [12]–[14], rosetta stone [15], co-evolution [16]–[18], network related methods [19]–[22], interologs [16], [23], protein interface analysis and docking [24]–[27] etc. Combining multiple prediction methods has been recently applied to predict PPI, for example, STRING (Search Tool for the Retrieval of Interacting Genes) [28], IBIS (Inferred Biomolecular Interactions Server) [29] and PIPS [30]. Although knowledge of interacting proteins is useful, researchers also require information about the mode of interaction. Then, the binding interface of PPI has been investigated by several kinds of methods from segments/motifs/domains (eg. ANCHOR, α-MoRF and PIPE-sites) [31]–[35], structural docking [36]–[38] to correlated mutations [39], [40].

It is virtually axiomatic that “sequence specifies structure”, which gives rise to an assumption that knowledge of the amino acid sequence alone might be sufficient to estimate the interacting propensity between two proteins for a specific biological function [41]. Accordingly, prediction of protein partners only based on sequence information is an ideal approach with rapidity and generality. Then, many efforts have been made on the sequence-based PPI prediction [42]–[44] and the use of this kind of methods is becoming increasingly widespread [5]. Inspired by this idea, we have developed a new method for PPI prediction only using the information of protein sequences [45]. This method was developed based on a new machine learning algorithm-support vector machine (SVM) combined with a newly designed kernel function and a conjoint triad feature for describing amino acids. The prediction ability of our approach is highly competitive in published sequence-based PPI prediction methods [46]. Herein, we developed a web-based tool, Sequence-based Protein Partners Search (SPPS), for high-throughput prediction of potential partners and networks for a query protein sequence. SPPS makes use of probability-based SVM method to screen possible protein partners from a series of protein databases covering several species. Furthermore, SPPS provides auxiliary analysis of potential protein partners in terms of some annotations. Therefore, SPPS may serve as a valuable tool to identify the possible interacting partners for a new protein with known sequence, or for an existing protein whose biological mechanism is unknown.

## Results

### Outline of SPPS server

**Figure 1:**
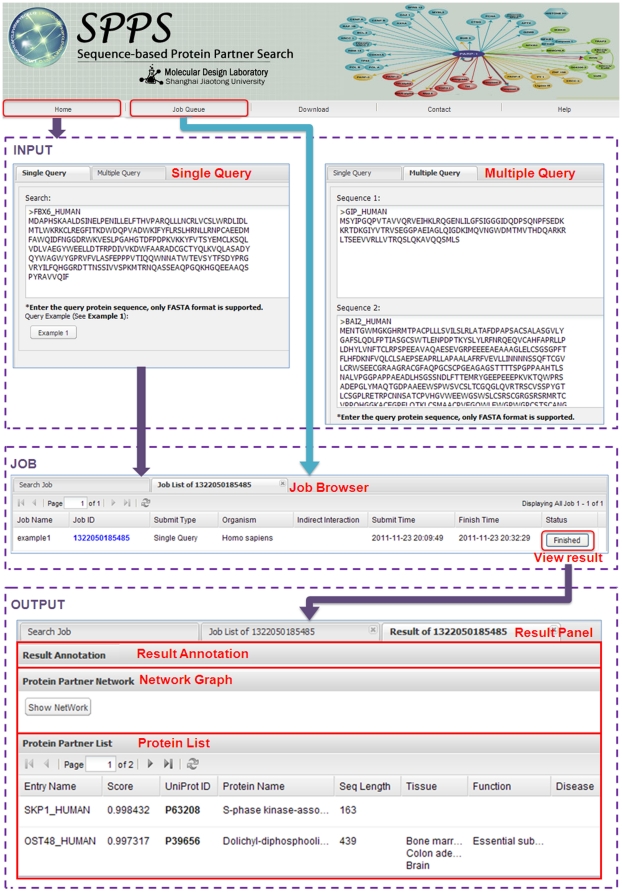
An overview of SPPS server.

The SPPS server consists of two parts, a front-end web interface written in Ajax framework ExtJS, with MySQL as the database system, and a back-end program “Kangaroo” for protein partners searching on a Linux Cluster server. The flowchart representation of the SPPS web server is shown in Figure 1. SPPS server provides two modes, “Single Query” mode and “Multiple Query” mode, to capture interacting partners for query protein. “Single Query” mode applies one query protein sequence to fish out its potential partners in a species-specific database. “Multiple Query” mode makes a rapid estimation of direct and indirect interactions between two query protein sequences. The web access is enabled via JBOSS webserver. Internet Explorer version 7 or above, Mozilla Firefox version 3.6 or above, Apple Safari and Google Chrome were thoroughly tested and thus recommended for SPPS.

### Availability and requirements

The input format of query protein sequences for SPPS server is the standard FASTA. The sequences of proteins can be either derived from in-house experiments, or directly taken from the databases embodying protein sequences, such as NCBI (http://www.ncbi.nlm.nih.gov), UniProt (http://www.uniprot.org/), and PIR (http://pir.georgetown.edu).

The models in SPPS server were originally built in 2006. Considering much data has been produced in the intervening time, these models were updated in the study using the latest collection of PPI data by Jan 2011. The statistical evaluation of all five models were calculated and shown in Table 1.

**Table 1:** The accuracies of prediction models constructed using our algorithm.

Species	Num. Seq	Num. PPIs^a^	5-CV^b^				“Single Query” Time (min)
			SE	SP	PRE	ACC	
*H.sapiens*	20027	39191	0.828	0.978	0.974	0.903	25
*C. elegans*	5070	4973	0.770	0.901	0.886	0.836	2
*D. melanogaster*	8767	22482	0.808	0.953	0.945	0.880	8
*S.cerevisiae*	14925	25064	0.851	0.979	0.976	0.915	10
*M.musculus*	15185	1225	0.802	0.882	0.872	0.842	4

a Known interactions for building classifier model, which were collected till Jan, 2011.

b The 5-CV performance of statistical learning methods can be measured by the quantity of true positives (TP), true negatives (TN), false positives (FP) and false negatives (FN). Precision [PRE = TP/(TP+FP)] is a measure of the accuracy provided that a specific class has been predicted. Accuracy [ACC = (TP+TN)/(TP+TN+FP+FN)] is another frequently used index for the overall classification performance, but it may be misleading as a result of highly unbalanced class distribution of used datasets. Sensitivity [SE = TP/(TP+FN)] and specificity [SP = TN/(TN+FP)] can assess a model's ability to correctly identify TP and TN, respectively, while they are usually interpreted in combination with each other.

The SPPS server is free to all users, including searching and access to known protein partners. After defining query protein sequence, two mandatory parameters must be set in order to submit a job: Specifying a “Job Name” enables the users to easily locate their queries in the “Job Queue” and selecting the “Organism” for the retrieval of potential partners from our protein repertoire against the query protein. Once the run is submitted, a transition window pops up with an associated Job ID. Each job submission is provided with unique Job ID based on the current date and time that serves as a permanent bookmarkable link to the data. The users can apply the unique Job ID or Job Name to track the progress of the calculation in the ‘Job Queue’ page of SPPS. Typical runs of ‘Single Query’ and ‘Multiple Query’ with ‘Consider indirect interaction’ option takes 2–25 minutes, depending on the number of protein repertoire in different species. The estimation of direct interaction in “Multiple Query” only takes several seconds. Upon completion of a job, a button labeled “Finished” emerges in the “Job Queue” page and can redirect the users to the result. In the future, we will update the list of proteins every 6 months in order to provide the latest receptors to screen.

As the result of ‘Single Query’, the output in SPPS is split into three main sections, namely, “Protein Partner List”, “Protein Partner Network” and “Result annotation”. “Protein Partner List” includes all predicted protein partners along with their overall confidence scores in probability. The confidence score is provided for each potential protein-protein interaction as described in the *Materials and Methods* and ranges from 0% to 100%, with 0% indicating maximum confidence for non-interaction and 100% indicating maximum confidence for interaction between two proteins. For example, a potential protein partner for a query protein with the estimated probability of 90% should be more likely to be the physical partner than one with a probability of 60%. Therefore, only potential partners of the query protein with probabilities larger than 50% are ranked as positives in descending order. If partners of a query protein have been predicted as positives, the potential interactive network from the query protein is constructed by integration of known PPIs, as shown in “Protein Partner Network”. In the current SPPS, candidate proteins from five species including “*Homo sapiens*”, “*Mus musculus*”, “*Caenorhabditis elegans*”, “*Drosophila melanogaster*”, and “*Saccharomyces cerevisiae*” are prepared to be fished out, each protein has been annotated with “Function”, “Disease”, “Tissue specificity”, “Interaction” and “Subcellular location”, and deposited in MySQL. User could check them from the result table. In addition, “Tissue Match” may select the predicted partners with the specific distribution from keywords by user input. In the “Known Interaction Match”, the experimental known partners of query protein in the predicted list can retrieve when user inputs the “Uniprot Entry Name” of the query protein (eg. “BRMS1_HUMAN”, “FBX6_HUMAN”). In addition, a download link is available for all known interactions of the query protein if “Uniprot Entry Name” of the query protein is submitted, which were collected from several PPI databases (eg. HPRD, STRING, DIP etc).

Contrary to “Single Query”, the output of “Multiple Query” mainly divides into “Direct Interaction Prediction” and “Indirect Interaction Prediction” sections. The probability score is always provided for direct PPI predication. One or two intermediate proteins linking two query proteins are also estimated if “Consider indirect interaction” option in “Multiple Query” mode is checked. All probabilities of prediction in the output are defined the same way as “Single Query”.

In addition to the “Search” option, SPPS also offers all training data, models and standalone software under its “Download” page, which facilitate users in their local machines if they have a great number of data to predict.

### Implementation

To test the reliability of the SPPS server, we searched for the interacting protein partners for human F-box protein 6 (FBXO6) using “Single Query” and predicted a few direct interactions not included in the training models from the latest literature among several species using “Multiple Query”.

FBXO6 protein is involved in the endoplasmic reticulum-associated degradation pathway by mediating the ubiquitination of glycoproteins. FBXO6 interacts with the innermost chitobiose in N-glycans of glycoprotein substrates by a small hydrophobic pocket in FBA domain and the introduction of point mutation into the residues in that pocket (FBXO6 null) impaired the binding activity toward its glycoprotein substrates [47]. In the study, candidates of human FBXO6 interacting partners were predicted using “Single Query” of SPPS server (Table 2) and top-five candidates were then tested by immunoprecipitation (IP) and western blot. The result showed that two of them, RPN1 (Ribophorin-1) and DDOST (Oligosaccharyl transferase 48 kDa subunit), are interacting partners of FBXO6 protein (Figure S1), suggesting that our computational models represent an effective means of protein-partner search and could be a useful tool for both basic PPI and advanced signal transduction studies.

**Table 2:** The top 10 potential protein partners of FBXO6 in human by SPPS “Single Query” search.

Rank	Probability	Gene Name	Accession no.	Protein Name
1	0.9984	SKP1_HUMAN	P63208	S-phase kinase-associated protein 1
2	0.9973	OST48_HUMAN	P39656	Dolichyl-diphosphooligosaccharide–protein glycosyltransferase 48 kDa subunit precursor
3	0.9957	RPN1_HUMAN	P04843	Dolichyl-diphosphooligosaccharide–protein glycosyltransferase subunit 1
4	0.9901	DDOST_HUMAN	B4DJE3	cDNA FLJ52929_highly similar to Dolichyl-diphosphooligosaccharide–proteinglycosyltransferase 48 kDa subunit
5	0.9876	IF4G2_HUMAN	P78344	Eukaryotic translation initiation factor 4 gamma 2
6	0.9869	HSP90B1_HUMAN	B4DHT9	Uncharacterized Protein
7	0.9851	TBG2_HUMAN	Q9NRH3	Tubulin gamma-2 chain
8	0.9846	DDX3Y_HUMAN	O15523	ATP-dependent RNA helicase DDX3Y
9	0.9819	SOS2_HUMAN	Q07890	Son of sevenless homolog 2
10	0.9814	HS90B_HUMAN	P08238	Heat shock protein HSP 90-beta

Besides“Single Query”mode, SPPS server also provides “Multiple Query” to quickly evaluate whether two proteins are directly and indirectly interactive based on the score of probability, which may help biologists to choose the most probable protein-protein interaction pairs for further experimental validation. Many directly interacting proteins from the latest literatures in 2011 [48]–[57], which were not included in the training models, have been successfully predicted by SPPS server, as listed in Table 3.

**Table 3:** Prediction of PPI not included in the models on variant species by using “Multiple Query” mode^a^.

No.^b^	Species	Protein 1	Protein 2	Probability
1 [48]	*H.sapiens*	GIP	BAI2	0.9735
2 [49]	*H.sapiens*	RASD1	EAR2	0.9243
3 [50]	*H.sapiens*	RELA	KEAP1	0.9999
4 [51]	*M.musculus*	TMM88	DVL2	0.9435
5 [52]	*M.musculus*	MTF1	SUMO1	0.8299
6 [53]	*M.musculus*	GRB2	mCAT1	0.8258
7 [54]	*C.elegans*	LST4	DYN1	0.9604
8 [55]	*S.cerevisiae*	GID9	GID2	0.9997
9 [56]	*S.cerevisiae*	HMO1	SPT6	0.9999
10 [57]	*D.melanogaster*	PSB1	PSB3	0.8891

a Protein1 and Protein2 represent two query proteins in “Multiple Query” mode respectively.

b Reference number for experiment validation.

SPPS server has been running since 2007 and is updated annually. Competitive prediction of our algorithm has been evaluated in 2010 among sequence-based PPI prediction methods [46]. Over 200 query protein sequences, including catalytic enzymes detected in biochemical or signal transduction experiments, a regulative factor in the lysosome, and a novel protein whose functions is unclear, have been screened. Five groups outside the authors' labs have become involved in screening. Therefore, the method could be helpful to more biologists when the server is open to publics.

## Discussion

By bringing together a protein-partner search engine and protein databases in a single program, the SPPS server is a convenient tool for the identification of potential protein partners of query proteins such as kinases, regulatory factors, and other components of much more complex protein machineries. This web server can also be used in constructing the signal transduction network for a known protein or a novel protein whose function is unknown. In general, one protein may interact with at least several partners including upstream and downstream regulators. As illustrated by the example of FBXO6, SPPS provides a good predictive ability for potential protein partner hits. These are useful guidelines for further experimental validation of signaling network around any given protein.

However, SPPS still has certain limitations, one of them being that the number of the models is not enough for covering all the species. The second one is that SPPS server has not considered interspecific interactions, such as the interactions between viral and human proteins, which may be vital in exploring targets responsible for infectious diseases. The running time for “Single Query” job is still slow due to large database and limited CPUs. To overcome these shortages, we are currently (i) collecting original interaction data produced by using the yeast two-hybrid based methods, mass spectrometry, protein chips and hybrid approaches to construct training models for more species, (ii) developing new kernel of SVM to adapt crossover interactions between different species, (iii) planning to provide more CPUs to accelerate the running process.

Discovering protein partners in large-scale network has been unprecedentedly appreciated due to the requirement to address the complicated process of biological systems by means of integrated technology. SPPS provides a new type of tool to facilitate the identification of direct or indirect protein partners and guides scientists to design new experimental directions. The SPPS server is available at a public web service http://mdl.shsmu.edu.cn/SPPS/.

## Methods

### Construction of the protein databases

SPPS requires a sufficient number of known protein sequences covering a diverse range of species. The protein sequences in our database were retrieved from UniProt (http://www.uniprot.org/), which is carried out by a Python script “Updater” at a 6-month interval. The database currently consists of more than 60,000 non-redundant protein sequences, with species covering “*Homo sapiens*”, “*Mus musculus*”, “*Caenorhabditis elegans*”, “*Drosophila melanogaster*”, and “*Saccharomyces cerevisiae*”, as shown in Table 1. In addition, the annotations for each protein in the database, such as subcellular location, tissue distribution, tissue specificity, known interactions, protein functions, and related disease were directly extracted from UniProt by Python script “Extractor”. These annotations were optionally used to refine the protein partner hits predicted by SPPS. For efficient analysis and management, all data are stored in a MySQL database (version 5.0).

### Probability estimation of protein partners using SVM

Our predictor is developed based on the estimation of PPI with SVM model. The details of the original algorithm have been published [45] and evaluated [46]. Five models including “*Homo sapiens*”, “*Mus musculus*”, “*Caenorhabditis elegans*”, “*Drosophila melanogaster*”, and “*Saccharomyces cerevisiae*” have been built based on the collected known PPIs with good accuracies (Table 1).

For the SPPS server, we further enhanced the algorithm by probability. Putative protein partners are ranked by the value of probabilities. Platt's approach was used to derive posterior probabilities for the estimated class membership *f(x_i_)* of observation *x_i_* [58]. A sigmoid function is fitted to all estimated *g(x_i_)* to derive probabilities by Eq. (1)
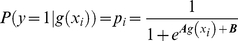
(1)where *A* and *B* are estimated by minimizing the negative log-likelihood of the training data, Eq. (2),
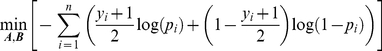
(2)The predictive probability ranges from 0% to 100%. In general, the higher the probability, the more accurate the prediction model presents.

### Immunoprecipitation (IP) and Western blot

293T cells transfected with either Flag-FBXO6 WT or Flag-FBXO6 Null were lysed in 6 ml of lysis buffer (50 mM Tris-HCl pH 7.5, 150 mM NaCl, 0.5% Nonidet P40, Roche complete EDTA-free protease inhibitor cocktail) for 20 min with gentle rocking at 4°C. Lysates were cleared using centrifugation (13,000 rpm, 10 min), the supernatant was subjected to immunoprecipitation (IP) with 50 µl of anti-FLAG M2 affinity resin (Sigma) overnight at 4°C with gentle inversion. Resin containing immune complexes was washed with 1 ml ice cold lysis buffer 4 times followed by three 1 ml Tris Buffered Saline (TBS) washes. Proteins were eluted with two 50 µl 150 µg/ml 3×Flag-peptide (Sigma) in TBS for 30 minutes, and the elutions were pooled for a final volume of 100 µl. Proteins in each elution were precipitated with cold acetone and the resulting pellet washed 2 times with cold acetone. Proteins were separated by 10% SDS-PAGE and transferred to NC membrane (Amersham Bioscience, Buckinghamshire, UK). After blocking with 5% nonfat milk in PBS, membranes were immunoblotted with indicated antibodies, followed by HRP-linked secondary antibodies (Cell Signaling). The signals were detected by SuperSignal West Pico Chemiluminescent Substrate kit (Pierce, Rockford, IL) according to manufacturer's instructions.

## Supporting Information

Figure S1 **Immunocomplexes from either 293T FBXO6^WT^ or 293T FBXO6^Null^ were immunoblotted with the indicated antibodies.** Both FBXO6^WT^ and FBXO6^Null^ interacted with Cullin1, only FBXO6^WT^ interacted with the DDOST and RPN1.
(TIF)
