# Supplementary figures and images for: SPPS: A Sequence-Based Method for Predicting Probability of Protein-Protein Interaction Partners
Source: PLoS One. 2012 Jan 26;7(1):e30938. doi: 10.1371/journal.pone.0030938 (PMC3266917; doi:10.1371/journal.pone.0030938)

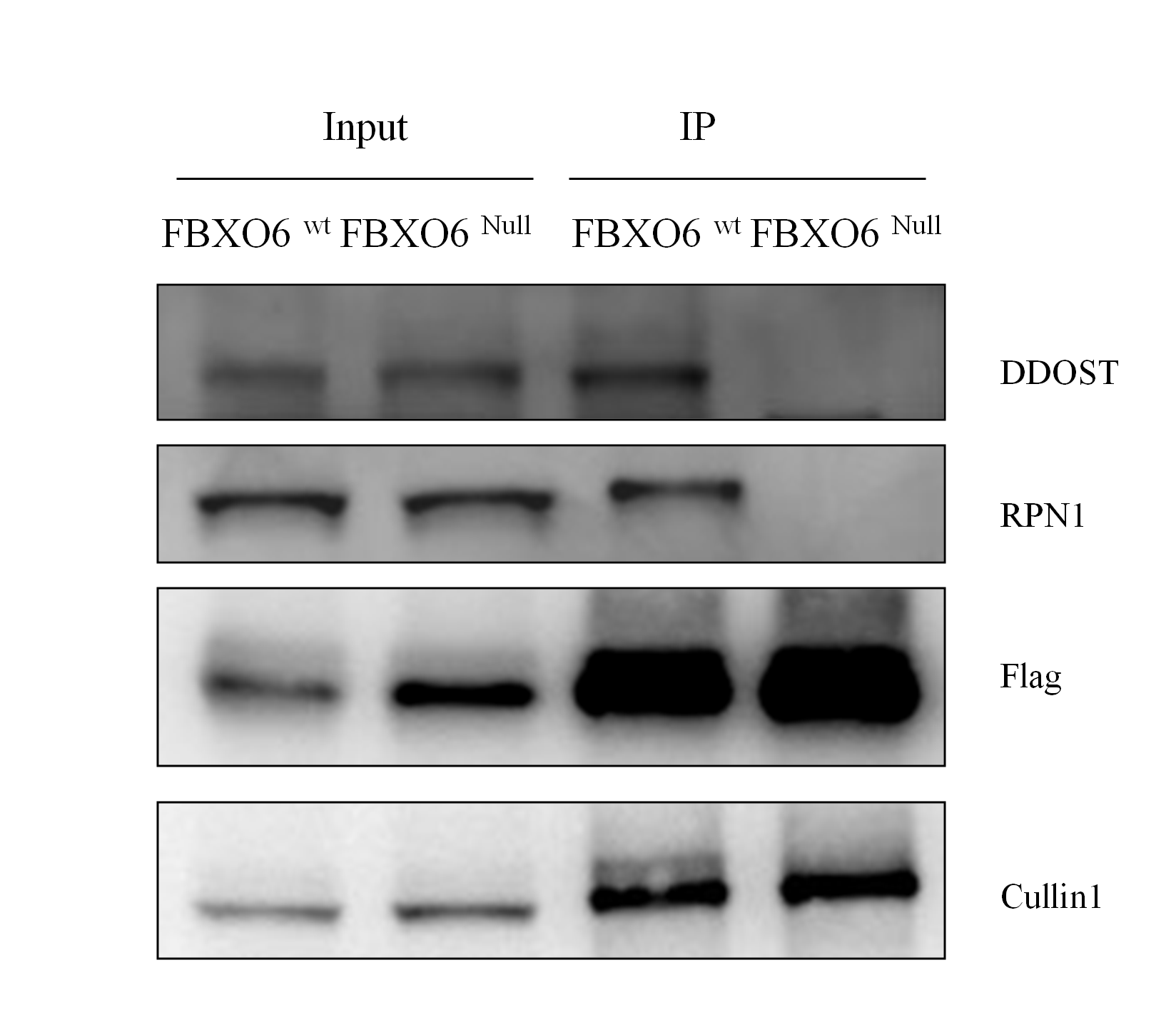

Supplement: Figure S1 — Immunocomplexes from either 293T FBXO6WT or 293T FBXO6Null were immunoblotted with the indicated antibodies. Both FBXO6WT and FBXO6Null interacted with Cullin1, only FBXO6WT interacted with the DDOST and RPN1. (TIF) [file pone.0030938.s001.tif]
